# A tissue‐equivalent phantom series for mammography dosimetry

**DOI:** 10.1120/jacmp.v5i4.1956

**Published:** 2004-11-24

**Authors:** William P. Argo, Kathleen Hintenlang, David E. Hintenlang

**Affiliations:** ^1^ U.S. Army Medical Department Center and School with duty at University of Florida 202 Nuclear Sciences Center, P.O. Box 118300 Gainesville Florida 32611‐8300 U.S.A.; ^2^ Robert Boissenault Oncology Institute Timber Ridge Medical Plaza 9401 SW Hwy 200, Bldg 800 Ocala Florida 34481 U.S.A.; ^3^ Department of Nuclear and Radiological Engineering 202 Nuclear Sciences Center, P.O. Box 118300 Gainesville Florida 32611‐8300 U.S.A.

**Keywords:** mammography, dose, phantoms

## Abstract

A breast tissue‐equivalent series (BRTES) of phantoms is manufactured to mimic both the attenuation and the density of the range of glandular and adipose tissue compositions encountered in mammography. The BRTES simulates breast tissues across the range of 20% to 70% glandularity and can be assembled in a variety of thicknesses to represent a compressed breast thickness corresponding to glandularity to simulate various patient demographics. The fabrication techniques are presented, and the physical properties of the completed series of phantoms are described. The BRTES phantoms provide a dosimetry comparison with commonly used phantoms, including the American College of Radiology accreditation phantom and BR12, a 50% glandularity tissue‐equivalent material. The comparison shows that the average glandular dose is a strong function of compressed breast thickness and tissue composition. Patient doses measured using photo‐timed exposures with the BRTES phantoms can be up to a factor of 3 greater than or less than the doses predicted using conventional phantoms.

PACS numbers: 87.52‐g, 87.57.‐s

## I. INTRODUCTION

### A. Mammography phantoms

Phantoms are important tools for the quality assurance of mammography equipment, evaluation of image quality, and accurate characterization of patient dose. The principle phantom used in mammography quality assurance is specified by the American College of Radiology (ACR).^(^ ^1^ ^)^ This phantom provides measures of image quality and is also used for dose measurements during physics testing. The latter provides a standardized measurement of an average glandular dose (AGD) that can be used to monitor the X‐ray tube output over time and compared to other mammography systems.

The use of the ACR accreditation phantom for dosimetry measurements must, however, be approached with caution, since it provides a quantitative prediction of dose for only a particular breast tissue composition and compressed thickness. This phantom is generally considered to be representative of an average compressed breast, of 4.2 cm thickness and composition represented by 50% fibroglandular and 50% adipose tissues, although we demonstrate that this is not a very good approximation. Local demographics may also lead to variations in the average compressed breast thickness and composition on a regional basis. Consequently, the AGDs measured using the ACR accreditation phantom are not representative of true patient doses.

The ACR accreditation phantom consists of 3.5 cm Lucite and a 0.7‐cm thick paraffin insert containing image quality indicators, resulting in an overall thickness of 4.2 cm. The radiological differences between the materials used in this phantom and breast tissue are magnified at the low X‐ray energies used in mammography, resulting in a response that can be quite different from realistic breast tissue compositions. Several other phantoms have been developed that more appropriately mimic the radiological properties of breast tissue. These are available from Nuclear Associates (Cleveland, OH) and CIRS (Norfolk, VA). The most common of these is known as BR12, originally developed by White^(^ ^2^ ^)^ to simulate a tissue composition of 50% fibroglandular tissue and 50% adipose tissue (i.e., 50% glandularity). CIRS also offers phantom materials of varying fractional tissue compositions and thicknesses.

We describe the construction of a series of tissue‐equivalent phantoms that can be constructed from readily available materials and use simple construction techniques. The techniques described here provide the ability for individual laboratories to manufacture high‐performance phantom materials that span the range of glandularities and compressed breast thicknesses of any patient population. We have refined and expanded the techniques described by White to develop homogeneous phantom materials that provide improved performance over a wide range of breast glandularities and thicknesses.

### B. Breast tissue composition

For radiological studies, the breast can be considered to be well approximated by a homogeneous mixture of fibroglandular and adipose tissues. The composition can therefore be uniquely described by the weight percent of fibroglandular tissue, the glandularity. The fibroglandular tissues comprise the tissues at risk for cancer development, and the dose to this tissue is the most important consideration in determining the radiation risks associated with mammography examinations. The fundamental compositions of both fibroglandular and adipose breast tissues are presented in ICRU 44.^(^ ^3^ ^)^

Glandularity is variable for individuals, but has been observed to correlate with compressed breast thickness^(^ ^4^ ^)^ and age.^(^ ^5^ ^)^ Both glandularity and breast thickness affect patient dose with general expectations of increased dose with increasing thickness (glandularity held constant) and increasing glandularity (thickness held constant). Variation of dose with thickness is evidenced through the increased peak kilovoltage (kVp) and milliampere seconds (mAs) required to maintain a constant image density when varying the thickness of phantom materials used in the routine testing of mammography automatic exposure control (AEC) modes. The quantitative variation with breast thickness and glandularity is not commonly evaluated, although it is important that both glandularity and thickness be accounted for when studying patient doses or optimizing imaging techniques for mammography.

Reviews of actual mammograms by Geise and Palchevsky^(^ ^4^ ^)^ have determined the glandular fraction to span a wide range with the average glandularity in their observed population to be 34% in contrast to the commonly assumed 50%. Helvie et al.^(^ ^6^ ^)^ reviewed compressed breast thickness and suggested the average compressed breast thickness is approximately 4.4 cm based on the 250 patients evaluated. Other studies^(^ ^5^ ^)^ demonstrate that compressed thickness and glandularity can be correlated with age, and anecdotal observations also frequently note significant variations in these parameters with patient demographics.

To reasonably predict the AGD for realistic patient populations, it is useful to utilize a series of phantoms that permit variation of both thickness and glandularity. We describe the construction of a breast tissue‐equivalent series (BRTES) of such phantoms that permit a more accurate evaluation of AGD by accommodating the differences in compressed breast thickness and glandularity that are expected for various patient populations. The BRTES phantoms are designed to accurately simulate glandularity from 0% to 100%, with phantoms specifically designed to match the range of clinically observed glandularity of 20% to 70%.

## II. MATERIALS AND METHODS

### A. Tissue‐equivalent materials

An epoxy resin matrix was selected as the basis for constructing a series of homogeneous phantoms representative of clinically observed glandularity and compressed thickness. Additional components were then selected that are readily incorporated into the matrix and ultimately produce the desired radiological properties. The desired phantom material must closely match the mass attenuation, mass‐energy absorption coefficients, and density of the known breast tissue over the mammography energy range. Material selection is additionally constrained by the need to produce a cost‐effective, user‐friendly, and low‐maintenance product from commercially available materials. The phantoms described can be easily fabricated from a mixture of readily available epoxy resins and chemical compounds.

XCOM Version 3.1, a software package written and developed by Hubbell^(^ ^7^ ^)^ at the Center for Radiation Research, National Bureau of Standards, is used to match the total mass attenuation coefficients between the proposed breast tissue substitutes and the adipose and glandular tissue properties reported in ICRU 44.^(^ ^3^ ^)^

The composite of materials that simulate glandular and adipose tissues were developed individually. Table 1 illustrates the selected material constituents of each tissue type by percent mass. By varying the ratios of these constituents, the desired physical properties and mass attenuation coefficients for the simulated glandular and adipose tissue material were matched to those predicted based on the tissue compositions reported in ICRU 44.^(^ ^3^ ^)^

**Table 1:** Tissue‐equivalent material composition by weight percent for 100% glandular and 100% adipose tissues

Materials	100% glandular	100% adipose
Araldite GY 60‐10 (epoxy resin)	49.43%	48.43%
Jeffamine T‐403 (hardener)	19.77%	19.37%
polyethylene powder (medium density)	18.50%	26.30%
phenolic microspheres	0.88%	1.20%
magnesium oxide powder	11.42%	4.70%

The BRTES phantoms were created to reproduce the observed correlations of compressed breast thickness and glandularity observed by Geise and Palchevsky.^(^ ^4^ ^)^ Once phantoms representing the attenuation properties and densities of 0% and 100% glandularity were obtained, they were combined in linear combinations of fractional weight percentage in order to provide BRTES materials of the desired glandularity.

#### A.1 Fabrication

The constituent materials for each desired BRTES composition were weighed and combined in the following order: epoxy resin (Araldite), polyethylene powder, magnesium oxide powder, phenolic microspheres, and hardener (Jeffamine). All items were thoroughly mixed by hand as they were added to the epoxy resin in order to ensure good homogeneity. Following the addition of hardener, the mixture was hand mixed for an additional 20 min. Hand mixing was used to prevent the introduction of air bubbles into the mixture; air bubbles compromise homogeneity, bulk density, and image quality. The mixture was poured into Teflon‐coated molds of approximately 1 cm depth, where curing took place for 48 h. Upon release from the mold, the phantom sections were finished and smoothed to the desired dimensions.

**Figure 1:**
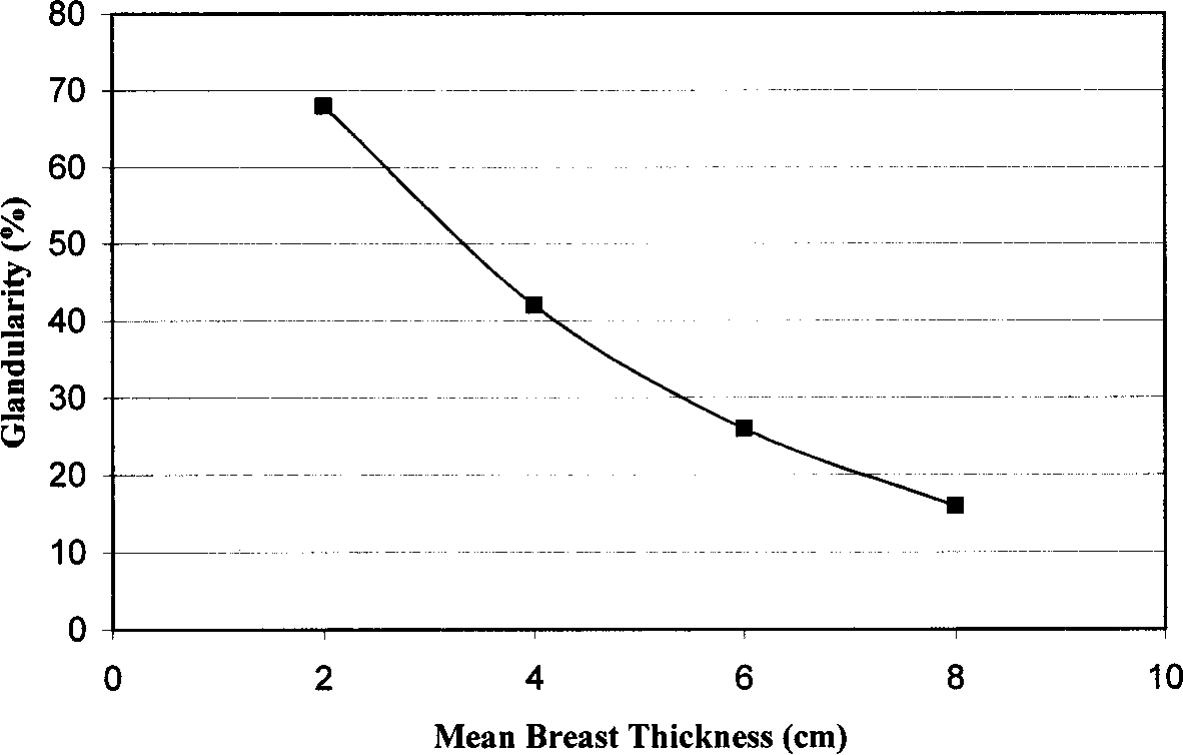
Correlation of glandularity and mean compressed breast thickness extracted from the demographic study by Geise and Palchevsky^(^ ^4^ ^)^

The BRTES phantoms were fabricated with lateral dimensions of 10.8 cm by 10.8 cm to facilitate dosimetry comparisons with the ACR phantom. Final thickness of the BRTES phantoms was based on the correlation of glandularity and thickness determined by Geise and Palchevsky^(^ ^4^ ^)^ and illustrated in Fig. 1 as the representative glandular/adipose composition as a function of mean breast thickness. A polynomial fit of the percent glandularity is used to predict the mean compressed breast thickness for any desired breast tissue composition. Geise and Palchevsky^(^ ^4^ ^)^ reported that the width of the glandularity distribution increases for compressed thickness between 3 cm and 7 cm but is narrower above and below this range. The phantoms were assembled from 1‐cm thick slabs of homogeneous BRTES material, with one slab in each phantom having a thickness an appropriate fraction of a centimeter to produce noninteger centimeter thicknesses when desired.

#### A.2 Characterization and dosimetry

The densities of the completed phantom sections were verified by geometrical and mass measurements, and the homogeneity was evaluated by mammography imaging. Mass attenuation and mass absorption coefficients for the final compositions were evaluated across the BRTES series using XCOM, based on the chemical composition of the prepared epoxy resin mixture. The elemental composition of the cured phantom materials is assumed to be equivalent to that of the prepared epoxy mixture, since the components are nonvolatile and form a highly viscous liquid that has the same mass prior to and after the curing process. A test to independently analyze the elemental composition of these materials has not been identified due to the physical properties and high hydrogen content of the simulant materials. The resulting mass attenuation and mass absorption coefficients are compared with those for the glandular and adipose tissue compositions provided by ICRU 44^(^ ^3^ ^)^ and those of White's BR12.^(^ ^2^ ^)^

Dosimetry measurements were made for the complete line of BRTES phantoms and compared to a parallel set of measurements performed on commercially available BR12 and ACR accreditation phantoms (Nuclear Associates). Measurements were performed on a General Electric Senographe DMR that was verified to perform within the ACR accreditation standards for kVp, half‐value layer (HVL), and AEC. A Keithley 35050A Dosimeter and 15 cm^3^ ion chamber was used to quantify exposure. Average glandular dose for each of the phantoms was evaluated across a range of energies using the standard measurement technique described by the ACR.^(^ ^1^ ^)^ In each case, the phantom being evaluated was used in the same physical location occupied by the ACR phantom when performing standard dose measurements.

The AGD was evaluated across the energy range most likely to be encountered for the particular combination of glandularity and compressed thickness being tested. Radiographic techniques were generated by selecting the desired kVp and permitting the automatic exposure control system to select the mAs. In order to convert the measured entrance air kerma to AGD for phantoms of varying glandularity, it is necessary to develop a more expansive set of dose conversion factors than those provided by the ACR.^(^ ^1^ ^)^ These factors were developed as a function of glandularity, thickness, kVp, and HVL based on Wu's^(^ ^8^ ^)^ Monte Carlo calculations. The dose conversion factors were interpolated using linearly weighted combinations of the dose conversion factors for 100% glandular, 50% glandular, and 100% adipose tissues presented by Wu.^(^ ^8^ ^)^ Calculated values are illustrated in Table 2 for the median thickness of population distribution thickness for each evaluated composition. Equation (1) can then be used to convert the air kerma to AGD:

(1) $$D_{g}=D_{gN}X$$

where $D_{g}$ is the average glandular dose, $D_{\text{gN}}$ is the average glandular dose per entrance air kerma, and *X* is the breast entrance air kerma.

**Table 2:** Average glandular dose (mGy) per entrance air kerma (mGy) for the BRTES phantom series

kVp	HVL	$D_{\text{gN}}\;(70\%)\;(1.86\;\text{cm})$	$D_{\text{gN}}$ (60%) (2.57 cm)	$D_{\text{gN}}\;(50\%)\;(3.36\;\text{cm})$	$D_{\text{gN}}\;(40\%)\;(4.2\;\text{cm})$	$D_{\text{gN}}\;(30\%)\;(5.39\;\text{cm})$	$D_{\text{gN}}\;(20\%)\;(6.96\;\text{cm})$
25	0.37	0.338	0.303	0.261	0.219	0.184	0.150
26	0.377	0.345	0.309	0.267	0.227	0.189	0.153
27	0.389	0.356	0.321	0.275	0.235	0.196	0.159
28	0.398	0.365	0.326	0.282	0.242	0.201	0.164
29	0.408	0.383	0.332	0.288	0.251	0.206	0.167
30	0.418	—	0.341	0.296	0.261	0.212	0.171

## III. RESULTS AND DISCUSSION

### A. Fabrication and characterization

The methods that were developed permitted the BRTES phantoms to be fabricated in a straightforward manner using readily available materials and simple laboratory techniques. By modifying some of the constituents originally proposed by White,^(^ ^2^ ^)^ we eliminated the need to mix the constituent materials under vacuum while still obtaining a series of homogeneous phantoms. The modified recipes also provide improved matching of the mass attenuation coefficients and mass absorption coefficients at typical mammography energies. The predicted mass attenuation coefficients of the glandular and adipose tissue substitutes developed agree with the ICRU 44^(^ ^3^ ^)^ values for 100% adipose and 100% glandular tissues within ±1.3% across the range of mammography energies, from 15 keV to 40 keV.

The density of each completed phantom was measured and compared to those reported by ICRU 46^(^ ^9^ ^)^ for average breast data across the full range of compositional mixtures. The comparison is detailed in Table 3.

**Table 3:** Comparison of densities of ICRU 46 and BRTES phantoms as a function of percent glandular composition

Phantom Composition % glandular	Measured Bulk Density $\left(g/{\mathrm{cm}}^{3}\right)$	ICRU 46 Average Adult Density $\left(g/{\mathrm{cm}}^{3}\right)$	% Difference
70.0%	1.029	0.999	3.00
60.0%	1.025	0.992	3.33
50.0%	0.993	0.985	0.81
40.0%	0.986	0.978	0.78
30.0%	0.984	0.971	1.34
20.0%	0.981	0.964	1.76

### B. Dosimetry

**Figure 2:**
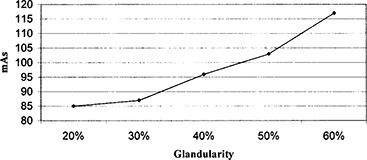
Photo‐timed mAs required for the varying glandularity of the BRTES phantoms at a constant thickness of 4.2 cm

The BRTES phantom series permits the measurement of AGDs that can more realistically reflect the patient dose received in mammography. Average glandular dose was measured for breast tissue across a full spectrum of glandularity and compressed thickness. Figure 2 illustrates the observed variation in photo‐timed mAs at 27 kVp for a series of 4.2 cm‐thick phantoms of differing glandular content. As expected, greater glandularity, representing denser tissue, results in increased photo‐timed mAs. However, it is clinically observed that increasing compressed breast thickness is correlated with a decrease in glandularity. This decreasing glandularity may be expected to offset the predicted increase in AGD with increasing compressed thickness to some degree.

**Figure 3:**
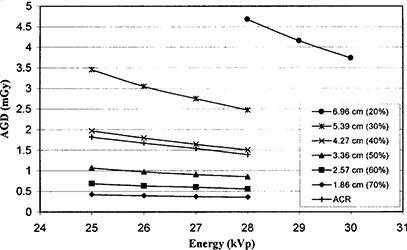
AGD vs kVp for BRTES phantoms representing compressed breast thicknesses of 1.86 cm, 2.57 cm, 3.36 cm, 4.27 cm, 5.39 cm, and 6.96 cm (corresponding to glandularity of 70%, 60%, 50%, 40%, 30%, 20%, respectively) to that predicted by the 4.2 cm‐thick ACR phantom

Dosimetry measurements show that AGD decreases for each phantom in the series with increasing kVp (Fig. 3). The results are quantitatively very similar to the changes in average dose as a function of kVp reported by LaVoy et al.^(^ ^10^ ^)^ for the ACR phantom. For this particular phantom, the dose is observed to decrease from 25 kVp to 28 kVp, with the 28 kVp dose being 77% of the 25 kVp dose. While similar reductions are observed for phantoms throughout the BTRES series, the magnitude of the dose changes dramatically with compressed thickness and composition. Table 4 illustrates that the percentage dose reduction from 25 kVp to 28 kVp is smallest for the high glandularity case (16% dose reduction) and greatest for the low glandularity case (29% dose reduction). The specific values of AGD represent the performance of this particular system. Other mammography systems would be expected to have similar trends in dose as a function of kVp and compressed breast thickness, although the quantitative values of AGD and the magnitude of differences as a function of compressed breast thickness and kVp are likely to differ due to variations in HVL, AEC adjustments, tube performance, etc.

**Table 4:** AGD (mGy) comparison of BRTES and ACR phantoms

Energy (kVp)	AGD for 70% (1.86 cm)	AGD for 60% (2.57 cm)	AGD for 50% (3.36 cm)	AGD for 40% (4.2 cm)	AGD for 30% (5.39 cm)	AGD for 20% (6.96 cm)	ACR (4.2 cm)
25	0.42	0.69	1.06	1.97	3.46	—	1.82
26	0.39	0.63	0.97	1.80	3.05	—	1.67
27	0.37	0.60	0.91	1.64	2.75	—	1.54
28	0.35	0.55	0.85	1.50	2.47	4.68	1.39
29	—	—	—	—	—	4.15	—
30	—	—	—	—	—	3.74	—

The results illustrate the significant differences that may be observed between patient doses and those estimated using the ACR phantom. Figure 3 illustrates that the 4.2 cm‐thick ACR phantom response is very similar to the 4.27 cm‐thick phantom of 40% glandularity. Since these phantom thicknesses are nearly identical, it appears that the ACR phantom more closely approximates a glandularity of slightly greater than 40%, as opposed to the commonly quoted 50% glandularity. The survey of Geise and Palchevsky^(^ ^4^ ^)^ showed the average patient breast in their study population was best simulated by a 34% glandularity, 16% less than the 50% glandularity assumed for the routinely used phantoms. These results have several implications for physicists evaluating the performance of mammography equipment and quantifying patient doses in mammography. It is quite clear that the ACR phantom does not accurately represent a breast of 50% glandularity as is commonly assumed, nor does it match the average glandularity observed in demographic studies.^(^ ^4^ ^,^ ^5^ ^)^ Table 4 and Fig. 3 show that the ACR phantom is likely to measure a dose of approximately 60% of the AGD delivered to the average patient.

Physicists frequently use BR12 to provide a better tissue substitute for the “average” breast of 50% glandularity. While BR12 does accurately mimic the radiological response of 50% glandular breast tissue, it is not likely to represent the average patient, who is likely to have a substantially lower glandularity.

## IV. CONCLUSION

The BRTES phantoms can be easily constructed in‐house and accurately mimic the attenuation and physical properties of breast tissue. The composition can be easily modified to provide phantoms of varying glandularity and geometry. The BRTES phantoms can provide the basis for readily characterizing patient doses for a variety of mammography systems.

The BRTES phantoms were compared with other commonly used phantoms and show that AGD rapidly increases as the compressed thickness increases and glandularity correspondingly decreases. The prediction of patient doses based on commonly used mammography phantoms (the ACR phantom and BR12) should be approached cautiously. Comparisons of the AGD measured for the ACR phantom was observed to vary from 0.3 times the dose delivered to a 20% glandularity (6.96 cm) phantom to 3.97 times the dose delivered to a 70% glandularity (1.86 cm) phantom. Dosimetry measurements made using the ACR phantom could therefore over‐ or underestimate some patient doses by a factor greater than 3. Average patients in the United States are likely to have less mean glandularity and greater compressed breast thickness than represented by the ACR and BR12 phantoms, with corresponding differences in dose. The commonly used thickness of 4 cm for both the ACR phantom and BR12 are likely to underestimate the AGD for most patients. The magnitude of the difference is a variable that is strongly dependent on local patient demographics. The BRTES phantom series provides a flexible set of tools that can be used to test realistic automatic exposure control operation and provide accurate quantification of patient dose for any set of observed patient demographics.

## ACKNOWLEDGMENTS

This research was supported in part by the U.S. Department of Energy, NEER Program, through Award DE‐FG07‐02ID14333.
